# Promoting Nerve Regeneration in a Neurotmesis Rat Model Using Poly(DL-lactide-**ε**-caprolactone) Membranes and Mesenchymal Stem Cells from the Wharton's Jelly: *In Vitro* and *In Vivo* Analysis

**DOI:** 10.1155/2014/302659

**Published:** 2014-07-10

**Authors:** T. Pereira, A. Gärtner, I. Amorim, A. Almeida, A. R. Caseiro, Paulo A. S. Armada-da-Silva, Sandra Amado, Federica Fregnan, A. S. P. Varejão, J. D. Santos, P. J. Bartolo, S. Geuna, A. L. Luís, A. C. Mauricio

**Affiliations:** ^1^Departamento de Clínicas Veterinárias, Instituto de Ciências Biomédicas de Abel Salazar (ICBAS), Universidade do Porto (UP), Rua de Jorge Viterbo Ferreira No. 228, 4050-313 Porto, Portugal; ^2^Centro de Estudos de Ciência Animal (CECA), Instituto de Ciências e Tecnologias Agrárias e Agro-Alimentares (ICETA), Rua D. Manuel II, Apartado 55142, 4051-401 Porto, Portugal; ^3^Departamento de Patologia e de Imunologia Molecular, Instituto de Ciências Biomédicas de Abel Salazar (ICBAS), Universidade do Porto (UP), Rua de Jorge Viterbo Ferreira No. 228, 4050-313 Porto, Portugal; ^4^Instituto Português de Patologia e Imunologia Molecular da Universidade do Porto (IPATIMUP), Rua Dr. Roberto Frias s/n, 4200-465 Porto, Portugal; ^5^Instituto Nacional de Saúde Doutor Ricardo Jorge (INSA), Rua Alexandre Herculano No. 321, 4000-055 Porto, Portugal; ^6^Centro para o Desenvolvimento Rápido e Sustentado de Produto (CDRsp), Instituto Politécnico de Leiria (IPL), Centro Empresarial da Marinha Grande, Rua de Portugal-Zona Industrial, 2430-028 Marinha Grande, Portugal; ^7^Faculdade de Motricidade Humana (FMH), Universidade Técnica de Lisboa (UTL), Estrada da Costa, 1499-002 Cruz Quebrada-Dafundo, Portugal; ^8^CIPER-FMH, Centro Interdisciplinar de Estudo de Performance Humana, Faculdade de Motricidade Humana (FMH), Universidade Técnica de Lisboa (UTL), Estrada da Costa, 1499-002 Cruz Quebrada-Dafundo, Portugal; ^9^UIS-IPL, Unidade de Investigação em Saúde da Escola Superior de Saúde de Leiria, Instituto Politécnico de Leiria, Morro do Lena, Alto do Vieiro, Apartado 4137, 2411-901 Leiria, Portugal; ^10^Neuroscience Institute of the Cavalieri Ottolenghi Foundation (NICO), Azienda Ospedaliero-Universitaria San Luigi Gonzaga, Regione Gonzole 10, Orbassano, 10043 Turin, Italy; ^11^Department of Clinical and Biological Sciences, University of Turin, 10126 Turin, Italy; ^12^Department of Veterinary Sciences, CIDESD, University of Trás-os-Montes e Alto Douro (UTAD), Quinta de Prados, 5001-801 Vila Real, Portugal; ^13^CEMUC, Departamento de Engenharia Metalúrgica e Materiais, Faculdade de Engenharia, Universidade do Porto, Rua Dr. Roberto Frias, 4200-465 Porto, Portugal; ^14^School of Mechanical, Aerospace and Civil Engineering & Manchester Institute of Biotechnology, The University of Manchester, Manchester M13 9PL, UK

## Abstract

In peripheral nerves MSCs can modulate Wallerian degeneration and the overall regenerative response by acting through paracrine mechanisms directly on regenerating axons or upon the nerve-supporting Schwann cells. In the present study, the effect of human MSCs from Wharton's jelly (HMSCs), differentiated into neuroglial-like cells associated to poly (DL-lactide-*ε*-caprolactone) membrane, on nerve regeneration, was evaluated in the neurotmesis injury rat sciatic nerve model. Results* in vitro* showed successful differentiation of HMSCs into neuroglial-like cells, characterized by expression of specific neuroglial markers confirmed by immunocytochemistry and by RT-PCR and qPCR targeting specific genes expressed.* In vivo* testing evaluated during the healing period of 20 weeks, showed no evident positive effect of HMSCs or neuroglial-like cell enrichment at the sciatic nerve repair site on most of the functional and nerve morphometric predictors of nerve regeneration although the nociception function was almost normal. EPT on the other hand, recovered significantly better after HMSCs enriched membrane employment, to values of residual functional impairment compared to other treated groups. When the neurotmesis injury can be surgically reconstructed with an end-to-end suture or by grafting, the addition of a PLC membrane associated with HMSCs seems to bring significant advantage, especially concerning the motor function recovery.

## 1. Introduction

Traumatic injuries affecting the central and the peripheral nervous system are often characterized by very limited recovery of lost functions and severe incapacity. In cases of no surgical treatment, spontaneous nerve regeneration is in many cases curtailed by scars, neuroma formation, mismatched nerve fascicles, or extensive splitting of the regrowing axons. Moreover, peripheral nerve damage is often associated with neuropathic pain, referred by patients as a more important reason for poor quality of life than the incomplete functional recovery [1]. Functional outcome is directly related with the degree of injury. Peripheral nerve regeneration is worse if a nerve gap exists, leading to functional impairment and frequently to neuroma [1, 2]. The time delay between the instant of traumatic nerve injury and of surgical repair is also an important factor determining functional outcome for various reasons [3].

Peripheral nerve neurotmesis is a relatively common type of traumatic injury affecting the peripheral nervous system. These constitute a severe nerve damage in which both nerve fibers and the nerve sheaths suffer disruption and spontaneous recovery becomes extremely difficult in cases when the peripheral nerve is not microsurgically reconstructed [4]. Whenever tension-free suturing is possible, direct end-to-end repair is the treatment of choice. However, when there is a nerve gap that resulted from the loss of the nerve tissue, an autologous nerve graft is typically undertaken, usually using an expendable sensory nerve, such as the sural nerve. However, autologous nerve grafting has important disadvantages, the most important being donor site morbidity that may lead to a secondary sensory deficit and occasionally neuroma and pain. In addition, no donor and recipient nerve diameters match often occurs and the fact of using, in most clinical situations, a sensory nerve to reconstruct a motor or a motor and sensory nerve might be the basis for poor functional recovery [5]. In some cases, entubulation can be used instead of grafting. Numerous experimental trials in animal models demonstrate the efficacy of tube-guides, made of different biomaterials, in supporting peripheral nerve regeneration. Some clinical cases also show that tube-guides can be safely employed in the reconstruction of peripheral nerves in human patients [6]. In these cases, the nerve will grow and regenerate from the proximal stump towards the distal nerve stump, while the ingrowth of fibrous tissue and neuroma formation are prevented by the tube-guide and simultaneously, a favorable microenvironment is created for the Wallerian degeneration and regeneration process during the healing period [6].

The development of cell-based therapies opened new venues in tissue regeneration including central and peripheral nerve system. Considering the peripheral nerve system, cellular systems are promising therapies to be applied alone or associated to scaffolds, especially, in neurotmesis injuries where the surgical reconstruction is not possible without tension and there is loss of tissue, creating critical defects of the nerve [6]. Regeneration is a physical process through which remaining tissues organize themselves to replace and repair injured or missing tissues* in vivo*. Stem cells from different sources are much likely the golden key for regenerative medicine [7]. Amongst stem cells, mesenchymal stem cells (MSCs) have become one of the most interesting targets due to their well-known characteristics. MSCs have a high plasticity and proliferative and differentiation capacity, together with promising immunosuppressive properties [6]. Furthermore, nowadays the identification and characterization of MSCs is well defined by recommendations and standards stated by the Mesenchymal and Tissue Stem Cell Committee of the International Society for Cellular Therapy (ISCT) [8]. The therapeutic effect of MSCs does not simply reside on their capacity to replace the original cells of damaged tissues. In fact, MSCs seem capable of secreting a variety of growth factors and cytokines that modify their microenvironment and induce the activity of endogenous progenitor cells within the injured tissue [9, 10]. Also, several studies demonstrate that MSCs exert a modulatory action on the inflammatory and immune responses and by these means contribute to tissue healing [11]. Therefore, the use of cellular systems is a rational approach for delivering growth-promoting factors and cytokines at the nerve lesion site [12]. MSCs can be isolated from several tissues, including bone marrow, skin, periosteum, amniotic fluid, umbilical cord blood and matrix, and adipose tissue [12]. Bone marrow represents the most frequently used tissue source of MSCs and these cells have been used in cell based therapies. However, as a source of MSCs, the bone marrow has several disadvantages, like limited number of MSCs available, the heterologous and nonconsistent nature of bone marrow preparations, and the possibility of donor site morbidity, as well as decreased number of MSCs along the adult life. For these reasons, it was important to identify alternative and more primordial MSCs sources that would allow a safe and controlled* ex vivo* expansion for potential allogeneic utilization [12]. Umbilical cord tissue-derived MSCs exhibit a neuronal phenotype [13–16] and are potentially useful for the treatment of neurodegenerative diseases [17, 18], again showing the versatility of this cell source. Interestingly, these cells are negative for the class II major histocompatibility complex (MHC) and have low expression of MHC class I [15], increasing their potential for MSC-based therapies. In addition, these cells represent a noncontroversial source of primitive mesenchymal progenitor cells that can be harvested after birth, cryogenically stored, thawed, and expanded for therapeutic uses [12, 19]. In the present work, the HMSCs isolated from the umbilical cord Wharton's jelly were tested concerning the nerve regeneration after a neurotmesis injury surgically reconstructed after an end-to-end and a graft procedure. Since these HMSCs are not only capable of differentiating into trilineage mesenchyme cell types, such as adipocytes, chondrocytes, and osteoblasts [8] but also capable of differentiating into neuronal-like cells, including astrocytes, oligodendrocytes, microglial, neurons, and neuroglial-like cells [12, 19], HMSCs and* in vitro* differentiated HMCs into neuroglial-like cells were used in this study. Morphology, immunocytochemistry, and DNA-based approaches were applied to sustain the identity of differentiated HMSCs into neuroglial-like cells. Regarding immunocytochemistry, antibodies reactive to growth associated protein 43 (GAP-43), reactive to glial fibrillary acidic protein (GFAP), and reactive to neuronal specific nuclear protein (NeuN) were used on slides with fixed HMSCs. As per DNA-based approaches, more particularly, RT-PCR and qPCR, the expression of seven genes, two housekeeping genes (*β*-actin and GAPDH), and five genes specific of neuronal cells (GFAP, NeuN, Nestin, NF-H, and GAP-43) were analyzed. Regarding the target genes, GFAP stands for glial fibrillary acidic protein, a protein present in the intermediate filament found in astroglial cells, cells that support and nourish neurons; NeuN stands for neuronal specific nuclear protein, a neuronal nuclear antigen commonly used as a biomarker for neurons; Nestin is a type VI intermediate filament (IF) protein expressed mostly in nerve cells where they are implicated in the radial growth of the axon; NF-H is the heaviest subunit of neurofilaments (NF) found in neurons, a major component of the neuronal cytoskeleton, and believed to function primarily to provide structural support for the axon and to regulate axon diameter; GAP-43 stands for growth associated protein 43, a protein expressed at high levels in neuronal growth cones during development, during axonal regeneration [19].

In order to implant cultured cells like HMSCs into defective nerves (with axonotmesis and neurotmesis injuries), there are two main techniques. The cellular system may be directly injected to the neural scaffold which has been interposed between the proximal and distal nerve stumps or around the crush injury (in neurotmesis and axonotmesis injuries, resp.). In alternative, implant can also be achieved by preadding the cells to the neural scaffold via injection or coculture (in most of the cellular systems, it is allowed to form a monolayer) and then the biomaterial with the cellular system is implanted in the injured nerve [12, 19]. In this experimental work a biomaterial commercially available (Vivosorb) to be used as a vehicle for the undifferentiated and differentiated cellular system tested in the nerve defects was used. Biomaterials are known to be able to support cellular systems to either differentiate into neuroglial-like cells or to enhance their paracrine effects on the overall regenerative process. Concomitantly, biomaterials can be directly involved in the regenerative process, helping to improve the motor and sensory functional recovery, shortening the healing period and avoiding regional muscular atrophy [20, 21]. A suitable biomaterial for nerve conduit must fulfill several biological and physicochemical requirements and might be of biological origin or synthetic. Such requirements include biocompatibility, biodegradability, permeability to ions and metabolites for the revascularization of the regenerated nerve, and biomechanical and surface properties that enable and modulate cellular systems adhesion [12, 19, 20]. Among synthetic biodegradable materials, poly(DL-lactide-*ε*-caprolactone) (PLC) attracted particular attention to our research group [21–25]. The biodegradation rate of PLC is estimated to be around 16 months and the degradation products of PLC are less acidic, like for instance poly(L-lactide): poly(glycolide) (PLGA), which may cause less damage to the surrounding tissue. Also, PLC is transparent facilitating the correct positioning of the nerve stumps. Previous* in vitro* studies have shown that PLC membranes and tube-guides are biocompatible with nerve cells and may facilitate nerve cell attachment, differentiation, and growth [21, 22, 26, 27]. Also,* in vivo* studies have demonstrated that PLC might improve morphological and functional recoveries, in axonotmesis and neurotmesis injuries of the rat sciatic nerve, while the structure of the polymer was still well preserved after 20 weeks in nerves repaired with PLC [21, 22]. Shin and colleagues (2009) tested PLC guiding-tubes and two different synthetic, bioabsorbable biomaterials and compared their efficacy for the reconstruction of the sciatic nerve with a reversal autograft. In this case, the PLC tube was, within the biomaterials, that with the best outcome, which was even comparable with the efficacy of the autograft [28]. In the present study the therapeutic value of undifferentiated human MSCs (HMSCs) isolated from the Wharton's jelly of the umbilical cord was tested* in vivo* or* in vitro* differentiated into neuroglial-like cells, together with a poly(DL-lactide-*ε*-caprolactone) (Vivosorb) membrane, to promote nerve regeneration in neurotmesis injuries surgically reconstructed with an epineural end-to-end suture or with an inverted autograft. A complete functional analysis was performed during the healing period of 20 weeks, including extensor postural thrust (EPT) and withdrawal reflex latency (WRL) tests, for evaluating the motor and nociception function, respectively. The functional assessment also included the kinematic analysis of the rat gait during the healing period. A morphometry analysis of the regenerated nerves was assessed at week 20. Also, the differentiation capacity of the HMSCs into neuroglial-like cells was tested* in vitro*, previously to the* in vivo* application.

## 2. Materials and Methods

### 2.1. Poly(DL-lactide-*ε*-caprolactone) (PLC) Membranes

**Figure 1:**
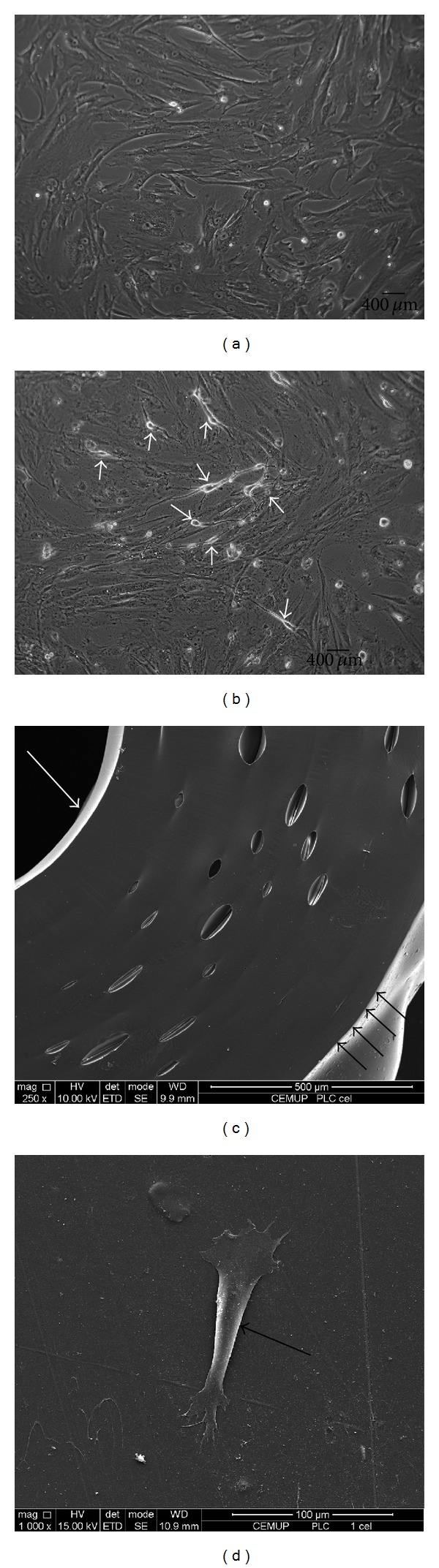
Monocultures of HMSCs from Wharton's jelly over PLC membrane exhibiting a mesenchymal-like shape with a flat polygonal morphology (a). Monocultures of HMSCs from Wharton's jelly over PLC membrane after 72 h of culture in neurogenic medium; differentiated HMSCs (small white arrows) became exceedingly long and there is a formation of typical neuroglial-like cells with multibranches (b) (magnification: 100x). SEM image of PLC tube-guide with inner diameter highlighted by large white arrow and outer diameter highlighted by small black arrows (magnification: 250x) (c). SEM image of HMSCs (large black arrow highlighting cell) cultured over a PLC disc (magnification: 1000x) (d).

Poly(DL-lactide-*ε*-caprolactone) (PLC) membranes (Vivosorb) were purchased from Polyganics BV, Groningen, Netherlands (FS01-006/20 Lot: FSA2009092311). Vivosorb is a flexible bioresorbable polymer film, made of poly(DL-lactide-*ε*-caprolactone) copolymer which presents retention of mechanical strength for up to 10 weeks throughout the critical healing period (Figures 1(c) and 1(d)).

### 2.2. Cell Culture and* In Vitro* Differentiation of HMSC from Wharton's Jelly Umbilical Cord

Human MSC from Wharton's jelly umbilical cord (HMSCs) were purchased from PromoCell GmbH (C-12971, lot-number: 8082606.7). This established human MSC cell line was preferred for* in vivo* testing in rats; since the number of MSCs obtained was higher in a shorter culture time, it was not dependent on donors availability and ethic committee authorization, and the protocol was much less time consuming which was advantageous for preclinical trials with a large number of experimental animals. Cryopreserved cells were cultured and maintained in a humidified atmosphere with 5% CO_2_ at 37°C. Mesenchymal stem cell medium (PromoCell, C-28010) was replaced every 48 hours. At 90% confluence, cells were harvested with 0.25% trypsin with EDTA (Gibco) and passed into a new flask for further expansion. HMSCs at a concentration of 2500 cells/mL were cultured and after 24 hours cells exhibited 30–40% confluence. Differentiation was induced with MSC neurogenic medium (PromoCell, C-28015). Medium was replaced every 24 hours during 3 consecutive days. The formation of neuroglial-like cells was observed after 24 hours in an inverted microscope (Zeiss, Germany) (Figures 1(a), and 1(b)).

HMSC cell line (differentiated) from Wharton's jelly was studied for cytogenetic analysis at passage 5. When confluence was reached, culture medium was changed and supplemented with 4 *μ*g/mL colcemid solution (stock solution, Cat. number 15212-012, Gibco). After 4 hours, HMSCs were collected and suspended in 8 mL of 0.075 M KCl solution supplemented with bovine fetal serum (BFS). Then the suspension was incubated in 37°C for 35 minutes. After centrifugation (1500 rpm), 8 mL of the fixative methanol : glacial acetic acid at 6 : 1 was added and mixed together, and the cells were again centrifuged. After 2 rounds of fixation, 2 new rounds were performed with the fixative methanol : glacial acetic acid at 3 : 1. After the last centrifugation, the HMSC suspension was spread onto very well glass cleaned slides. Analysis was performed by one scorer on Giemsa-stained cells.

Intracellular free Ca^2+^ concentration ([Ca^2+^]_*i*_) was measured in Fura-2-loaded cells by using dual wavelength spectrofluorometry as previously described [12, 19, 23, 26]. The measurements were performed on undifferentiated HMSCs after confluence was obtained and on neuroglial-differentiated HMSCs, cultured on Vivosorb discs in order to correlate the HMSCs ability to differentiate and survival capacity in the presence of the Vivosorb membrane (Figure 1(d)).

### 2.3. Immunocytochemistry

**Figure 2:**
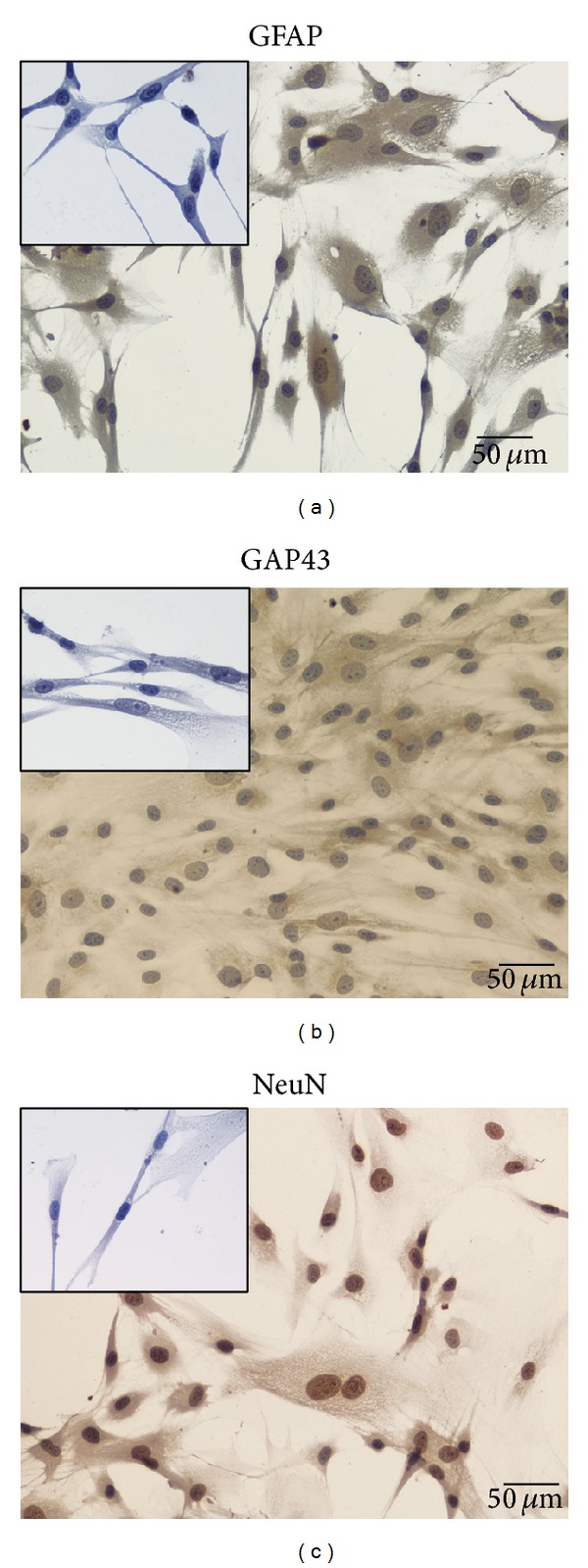
Neuroglial-like cells obtained from HMSCs* in vitro* differentiated with neurogenic medium exhibiting a positive staining for (a) GFAP which is a glial cell marker; (b) GAP-43 which is related with axonal outgrowth; and (c) NeuN which is a marker for nucleus of neurons. Undifferentiated HMSC cells from the Wharton's jelly presenting a negative staining for (small panel inserted in (a)) GFAP; (small panel inserted in (b)) GAP-43, and (small panel inserted in (c)) NeuN (magnification: 200x).

At passage 3, HMSCs were trypsinized, washed, and resuspended in mesenchymal stem cell medium (PromoCell, C-28010) at a concentration of 1 × 10^5^ cells/mL. HMSCs were fixed with paraformaldehyde at 4°C for 15 min and washed with distilled water before permeabilization in 0.5% Triton-X100. Non-specific binding was blocked using blocking solution (PBS containing 1% bovine serum albumin (BSA)) for 1 hour at room temperature. HMSCs were then incubated 2 hours at room temperature with primary antibodies from rabbit against antigrowth associated protein-43 (GAP-43, 1 : 200) (Chemicon, AB5220) and against antiglial fibrillary acidic protein (GFAP, 1 : 500) (Chemicon, AB5804) and from mouse against antineuronal nuclei (NeuN, 1 : 100) (Chemicon, MAB377). After washing, HMSCs were incubated 15 minutes with goat anti-rat IgG (Millipore, AP136P) and goat anti-rabbit IgG (Millipore, 12-348MN) secondary antibodies. After several washes in PBS, HMSCs were incubated with horseradish peroxidase (HRP-) coupled streptavidin for 10 min. DAB (diaminobenzidine) served as chromogen (Figure 2).

### 2.4. Reverse Transcriptase Polymerase Chain Reaction (RT-PCR)

Reverse transcriptase Polymerase chain reaction (RT-PCR) and qPCR targeting specific genes expressed by neuronal cells were performed. For that, primers were designed targeting seven human genes based on the literature [29–31]. DNA sequences from GAP-43, NF-H, Nestin, GAPDH, *β*-actin, NeuN, and GFAP genes from mice (*Mus musculus*), rat (*Rattus norvegicus*), and human (*Homo sapiens*) were downloaded from GenBank (http://www.ncbi.nlm.nih.gov/genbank) and aligned using the Clustal Omega bioinformatic tool from EMBL-EBI (http://www.ebi.ac.uk/Tools/msa/clustalo). The primers targeting the human genes are listed in Table 1. Both differentiated and undifferentiated HMSC's cultures were harvested with 0.25% trypsin EDTA solution (Gibco) and centrifuged at 2000 rpm 4°C during 5 min. Cell pellets were used for total RNA extraction using an adequate extraction kit, High Pure RNA Isolation kit (Roche). Briefly, cell pellets were lysed with a lysis buffer and loaded into a High Pure Filter Tube; DNA was removed with DNase I enzyme, washed twice on column, and eluted with 100 *μ*L of Elution Buffer. RNA was quantified and its quality was assessed by using a nanodrop ND-1000 spectrophotometer and reads from 220 nm to 350 nm, and then stored at −80°C until further use. In the following step, cDNA was synthesized from the purified RNA. To fulfill that issue, the kit Ready-To-Go You-Prime First-Strand Beads (GE Healthcare) was used following the manufacturer instructions. Briefly, 1.5 *μ*g of total RNA was used and diluted in DEPC-treated water to a 30 *μ*L final volume in a RNase-free microcentrifuge tube, then heated at 65°C for 10 minutes, and then chilled in ice; transfer the RNA solution to the kit tube containing the first-strand reaction mix beads; add 0.2 *μ*g of Oligo(dT) primer and DEPC-treated water to a 33 *μ*L final volume; mix the content and incubate at 37°C for 60 minutes. cDNA was synthesized and stored at −20°C until further use. Of referring that, due to the use of the Oligo(dT) primer, the synthesized cDNA corresponds to the mRNA present in the sample at the time of collection. cDNA synthesized from undifferentiated and differentiated HMSCs was used to check the expression of seven genes, two housekeeping genes (*β*-actin and GAPDH) and five specific of neuronal cells (GFAP, NeuN, Nestin, NF-H, and GAP-43). As previously described, primers were designed* in house* and then synthesized in an external laboratory (MWG Operon, Germany). Upon arrival, primers were rehydrated in DNase/RNase free water in a concentration of 100 pmol/*μ*L. Quantitative PCR (qPCR) was performed in a iCycler iQ5 (BioRad) apparatus using the iQ SYBR Green Supermix (BioRad). Each pair of primers targeting a gene was used to analyze its expression in the differentiated and undifferentiated HMSC's cDNA, in triplicate, along with a negative control. The plates containing the mix targeting the seven genes for both types of cells were submitted to the following cycles of temperatures: 95°C during 4 minutes, 35 cycles comprising 95°C during 20 seconds, 55°C during 20 seconds, and 72°C during 20 seconds ending with real-time acquisition and final extension of 75°C for 7 minutes. After cycling temperatures, the number of cycle threshold for each well was recorded. The plate containing the amplified genes or qPCR products was kept in ice and observed in a 2% agarose gel to check and reinforce the identity of the amplicons. Briefly, 2 gr of NuSieve 3 : 1 Agarose (Lonza) was mixed with 100 mL Tris-Acetate-EDTA buffer, melted, mixed with ethidium bromide in a final concentration of 0.2 *μ*g/mL, and loaded in a horizontal electrophoresis apparatus. After solidification, 15 *μ*L of the qPCR products was loaded in the agarose wells and submitted to a 120 V potential difference during 40 minutes to separate the amplicons. Gel was then observed under UV light and pictures were recorded using the GelDoc 2000 (BioRad) and Quantity One software (BioRad).

### 2.5. Surgical Procedure

All animal testing procedures were carried out in conformity with the Directive 2010/63/EU of the European Parliament and with the approval of the Veterinary Authorities of Portugal in accordance with the European Communities Council Directive of November 1986 (86/609/EEC). Humane end points were followed in accordance to the OECD Guidance Document on the Recognition, Assessment, and Use of Clinical Signs as Humane Endpoints for Experimental Animals Used in Safety Evaluation (2000). For the* in vivo* testing, Sasco Sprague adult rats (Charles River Laboratories, Barcelona, Spain) were divided into groups of 6 animals each. All animals were housed in a temperature and humidity controlled room with 12-12 hours light/dark cycles, two animals per cage (Makrolon type 4, Tecniplast, VA, Italy), and were allowed normal cage activities under standard laboratory conditions. The animals were fed with standard chow and water* ad libitum*. Adequate measures were taken to minimize pain and discomfort taking in account human endpoints for animal suffering and distress. Animals were housed for two weeks before entering the experiment. For surgery, animals were placed prone under sterile conditions and the skin from the clipped lateral right thigh scrubbed in a routine fashion with antiseptic solution. The surgeries were performed under an M-650 operating microscope (Leica Microsystems, Wetzlar, Germany). Under deep anesthesia (ketamine 90 mg/Kg; xylazine 12.5 mg/Kg, atropine 0.25 mg/Kg i.m.), the right sciatic nerve was exposed through a skin incision extending from the greater trochanter to the midthigh distally followed by a muscle splitting incision [22]. The right sciatic nerve transection (neurotmesis) injury was performed, immediately above terminal nerve ramification, with a straight microsurgical scissor. A group of 6 animals was used as control and with the sciatic nerve being left intact (Group 1:* Control*). In Group 2 the sciatic nerve was transected and left unrepaired, with the nerve stumps sutured to surrounding tissue in order to prevent nerve regeneration (Group 2:* Gap*). In Group 3, immediate cooptation with 7/0 monofilament nylon epineural sutures of the 2 transected nerve endings was performed (Group 3:* End-to-End*). In Group 4, the two endings of nerve transection were immediately sutured with a 7/0 monofilament nylon suture and enwrapped in a PLC (Vivosorb) membrane covered with a monolayer of nondifferentiated HMSCs (Group 4:* End-to-EndPLCCellnonDif*); in Group 5, the two endings of nerve transection were immediately sutured as the previous group and enwrapped in a PLC (Vivosorb) membrane covered with a monolayer of differentiated HMSCs (Group 5:* End-to-EndPLCCellDif*). In the last three groups, the sciatic nerve was bisected immediately above the terminal nerve ramification and at a 10 mm distal point. The resulting nerve graft, with a length of 10 mm, was inverted 180° and sutured with 7/0 monofilament nylon. One group was used as control for the graft (Group 6:* Graft*); in another group the graft was enwrapped in a PLC (Vivosorb) membrane covered with a monolayer of non-differentiated HMSCs (Group 7:* GraftPLCCellnonDif*) and in the last of the groups, the graft was enwrapped in a PLC (Vivosorb) membrane covered with a monolayer of differentiated HMSCs (Group 8:* GraftPLCCellDif*) (Figures 1(a), 1(b), 1(c), and 1(d)). No local or systemic signs of rejection or foreign body were observed in the experimental animals transplanted with PLC membranes and HMSCs (undifferentiated and differentiated). No immunosuppressive treatment was given to any of the experimental animals during the entire study.

### 2.6. Functional Assessment

All animals were tested preoperatively (week 0) and every week until week 12 and every 2 weeks until the end of follow-up time (20 weeks). Animals were gently handled and tested in a quiet environment to minimize stress levels.

#### 2.6.1. Motor Performance and Nociceptive Function

The extensor postural thrust (EPT) was originally proposed by Thalhammer and collaborators, in 1995 [32] as a part of the neurological recovery evaluation in the rat after sciatic nerve injury. For this test, the entire body of the rat, excepting the hind-limbs, was wrapped in a surgical towel. Supporting the animal by the thorax and lowering the affected hind-limb towards the platform of a digital balance, elicits the EPT. As the animal is lowered to the platform, it extends the hind-limb, anticipating the contact made by the distal metatarsus and digits. The force in grams (g) applied to the digital platform balance (model TM560; Gibertini, Milan, Italy) was recorded. The same procedure was applied to the contralateral, unaffected limb. Each EPT test was repeated 3 times and the average result was considered. The normal (unaffected limb) EPT (NEPT) and experimental EPT (EEPT) values were incorporated into (1) to derive the functional deficit (varying between 0 and 1), as described by Koka and Hadlock, in 2001 [33]. Consider

(1) $$\begin{array}{c} \text{Motor}\;\text{Deficit}=\frac{\text{NEPT}-\text{EEPT}}{\text{NEPT}}. \end{array}$$

To assess the nociceptive withdrawal reflex (WRL), the hotplate test was modified as described by Masters and collaborators [34]. The rat was wrapped in a surgical towel above its waist and then positioned to stand with the affected hind paw on a hot plate at 56°C (model 35-D, IITC Life Science Instruments, Woodland Hill, CA). WRL is defined as the time elapsed from the onset of hotplate contact to withdrawal of the hind paw and measured with a stopwatch. Normal rats withdraw their paws from the hotplate within 4.3 s or less [35]. The affected limbs were tested 3 times, with an interval of 2 min between consecutive tests to prevent sensitization and the three latencies were averaged to obtain a final result [36]. If there was no paw withdrawal after 12 s of stimulation, the heat stimulus was removed to prevent tissue damage, and the animal was assigned the maximal WRL of 12 s [37, 38].

#### 2.6.2. Kinematic Analysis

Ankle kinematics was carried out before nerve injury (week 0), and at the 20-week follow-up time. Animals walked on a Perspex track with length, width, and height of, respectively, 120, 12, and 15 cm. In order to ensure locomotion in a straight direction, the width of the apparatus was adjusted to the size of the rats during the experiments. The rats' gait was video recorded at a rate of 300 images per second (CASIO EXILIM PRO EX-F1, Japan). The camera was positioned at the track's half-length where gait velocity was steady and 1 m distant from the track obtaining a visualization field of 14 cm wide. The video images were stored in a computer hard disk for latter analysis using an appropriate software APAS (Ariel Performance Analysis System, Ariel Dynamics, San Diego, USA). 2D biomechanical analysis (sagittal plan) was carried out applying a two-segment model of the ankle joint, adopted from the model firstly developed by [24]. The rats' ankle angle was determined using the scalar product between a vector representing the foot and a vector representing the lower leg. With this model, positive and negative values of position of the ankle joint (*θ*°) indicate dorsiflexion and plantarflexion, respectively. For each step cycle the following time points were identified, initial contact (IC), opposite toe off (OT), and heel rise (HR) and toe-off (TO) [24, 39, 40], and were time normalized for 100% of step cycle. The normalized temporal parameters were averaged over all recorded trials. A total of six walking trials for each animal with stance phases lasting between 150 and 400 ms were considered for analysis, since this corresponds to the normal walking velocity of the rat (20–60 cm/s) [24, 41, 42].

### 2.7. Histology and Scanning Electron Microscopy (SEM)

Nerve samples (10-mm-long sciatic nerve segments distal to the crush site and from un-operated controls) were processed for histological analysis of myelinated nerve fibers [43]. Fixation was carried out using 2.5% purified glutaraldehyde and 0.5% saccarose in 0.1 M sorensen phosphate buffer for 6–8 hours and resin embedding was carried out following Glauerts' procedure [44]. Series of 2 *μ*m thick semithin transverse sections were cut using a Leica Ultracut UCT ultramicrotome (Leica Microsystems, Wetzlar, Germany) and stained by Toluidine blue. Histological observation was carried out on a DM4000B microscope equipped with a DFC320 digital camera and an IM50 image manager system (Leica Microsystems, Wetzlar, Germany).

Prior to scanning electron microscopy (SEM) analysis, the HMSCs cultured on PLC discs and the PLC tube-guide without HMSCs Figures 1(c) and 1(d) were first fixed with 1.5% glutaraldehyde in 0.14 M sodium cacodylate buffer (pH 7.3) for 2 h at 4°C. Afterwards, the PLC samples with and without the HMSCs were dehydrated using graded ethanol solutions from 60% to 100%, 5 minutes each, and subjected to critical point drying. Finally, the samples were mounted on aluminum stubs using double-side adhesive tape and sputter coated with gold/palladium thin film, using the SPI Module Sputter Coater equipment for 100 seconds and with a 15 mA current. The SEM/EDS exam was performed using a high resolution (Schottky) Environmental Scanning Electron Microscope with X-Ray Microanalysis and Electron Backscattered Diffraction analysis: Quanta 400 FEG ESEM/EDAX Genesis X4M.

### 2.8. Statistical Analysis

Data was analysed using two-way mixed factorial ANOVA (General Linear Model). The design included two between-subjects variables with two conditions or levels (grafting versus nongrafting) and three conditions (no-cells, undifferentiated cells, and differentiated cells) and one within-subjects factor that included the time-repeated functional measures. Mauchly's test was used to assert sphericity and, if necessary, degrees of freedom correction was introduced using the Greenhouse-Geisser's epsilon. Simple planned contrasts (General Linear Model, simple contrasts) were used to compare data pooled across all experimental groups during recovery against preoperative data. Pairwise comparisons between groups were carried out by the HSD Tukey's test. All statistical procedures were performed with the statistical package SPSS (version 17.0, SPSS, Inc). Data is presented as mean ± standard deviation of the mean (SD) or as mean ± standard error of the men (SEM).

## 3. Results

### 3.1. Confirmation of HMSCs Differentiation into Neuroglial-Like Cells by Immunocytochemistry and Karyotype Analysis

The phenotype of HMSCs was assessed by PromoCell. Rigid control of quality tests was performed for each PromoCell's lot of HMSCs. HMSCs were tested for cell morphology, adherence rate and viability. Furthermore, each cell lot was characterized by flow cytometry analysis for a comprehensive panel of markers, such as PECAM (CD31), HCAM (CD44), CD45, and Endoglin (CD105). The HMSCs exhibited a mesenchymal-like shape with a flat and polygonal morphology. During expansion the cells became long spindle-shaped and colonized the whole culturing surface (Figure 1(a)). After 72 hours of culture in neurogenic medium, we observed a morphological change. The cells became exceedingly long and there was a formation of typical neuroglial-like cells with multibranches and secondary branches (Figure 1(b)). Initially, the testing of differentiation into MSCs neuroglial-like cells was based on the expression of typical neuronal markers by immunocytochemistry, such as glial fibrillary acidic protein (GFAP), growth-associated protein (GAP)-43, and neuronal specific nuclear protein (NeuN). Undifferentiated HMSCs were negatively labeled for GFAP, GAP-43, and NeuN (inserted panels in Figures 2(a), 2(b), and 2(c)). After 72 hours of differentiation, HMSCs became positively stained for GFAP (Figure 2(a)) and GAP-43 (Figure 2(b)), and all nuclei of neuroglial-like cells were also positive for NeuN, demonstrating successful differentiation of HMSCs into neuroglial-like cells (Figure 2(c)).

Undifferentiated HMSCs exhibited a normal star-like shape with a flat morphology (Figures 1(a) and 1(d)). After* in vitro* differentiation, HMSCs morphology changed into typical neuroglial-like pattern with multibranches and secondary branches (Figure 1(b)). Giemsa-stained cells of differentiated HMSC cell line at passage 5 were analyzed for cytogenetic characterization. However, no metaphases were found; therefore, the karyotype could not be established. However, the karyotype of undifferentiated HMSCs was determined previously and no structural alterations were found demonstrating absence of neoplastic characteristics in these cells, as well as chromosomal stability to the cell culture procedures [45].

### 3.2. Confirmation of HMSCs Differentiation into Neuroglial-Like Cells by RT-PCR Analysis

Both differentiated and undifferentiated HMSCs were harvested and their RNA purified and converted to cDNA using adequate procedures. Primers targeting typical neuronal markers, two housekeeping genes (*β*-actin and GAPDH), and five specific of neuronal cells (GFAP, NeuN, Nestin, NF-H and GAP-43) were used to support the differentiation into MSCs neuroglial-like cells (Table 1).

**Figure 3:**
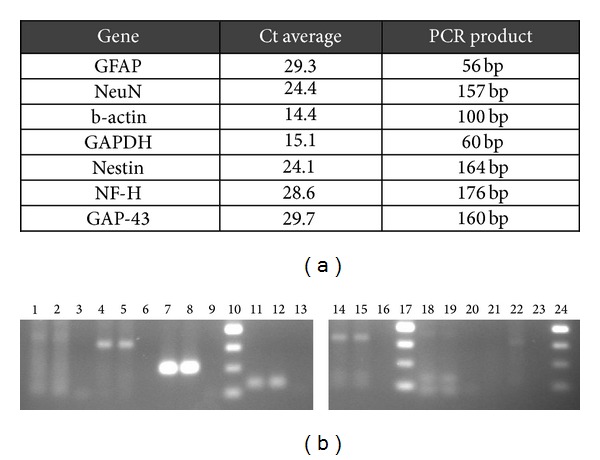
In this figure average of Ct values (a) and agarose gel profile of the amplification of the selected genes from the undifferentiated HMSCs (b) are shown. Agarose gel of the undifferentiated HMSCs' gene expression: lane 1 to 3, duplicates of GFAP gene and negative; lane 4 to 6, duplicates of NeuN gene and negative; lane 7 to 9, duplicates of b-actin gene and negative; lane 10, 50 bp DNA ladder; lane 11 to 13, duplicates of GAPDH gene and negative; lane 14 to 16, duplicates of Nestin gene and negative; lane 17, 50 bp DNA ladder; lane 18 to 20, duplicates of NF-H gene and negative; lane 21 to 23, duplicates of GAP-43 gene and negative; lane 24, 50 bp DNA ladder.

In the following (Figure 3), the average of Ct values and the agarose gel of the PCR products from experiments over the undifferentiated HMSCs is shown. In these cells, the molecular analysis showed a very small amplification of GFAP gene, absence of amplification of the NF-H and GAP-43 genes, and reasonable amplification of NeuN, *β*-actin, GAPDH, and Nestin genes. Amplification of a given gene is correlated with its expression seeing that the template DNA is the one generated from mRNA.

**Figure 4:**
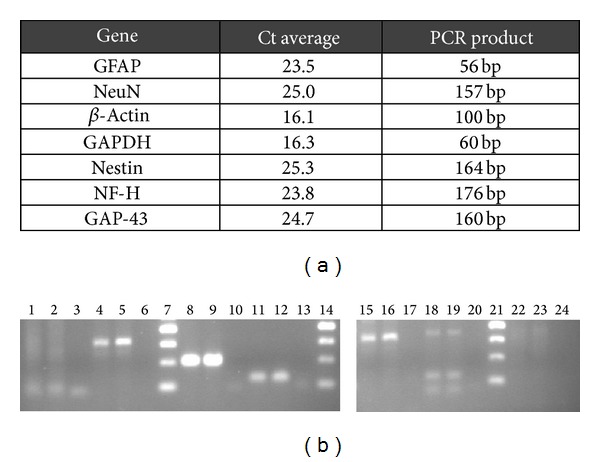
In this figure the average of Ct values (a) and agarose gel profile of the amplification of the selected genes from the differentiated HMSCs (b) are shown. Agarose gel of the gene expression of differentiated HMSCs: lane 1 to 3, duplicates of GFAP gene and negative; lane 4 to 6, duplicates of NeuN gene and negative; lane 7, 50 bp DNA ladder; lane 8 to 10, duplicates of *β*-actin gene and negative; lane 11 to 13, duplicates of GAPDH gene and negative; lane 14, 50 bp DNA ladder; lane 15 to 17, duplicates of Nestin gene and negative; lane 18 to 20, duplicates of NF-H gene and negative; lane 21, 50 bp DNA ladder; lane 22 to 24, duplicates of GAP-43 gene and negative.

In Figure 4, the same results are shown now for the differentiated HMSCs. In this case, the molecular analysis shows a similar amplification for NeuN, *β*-actin, GAPDH, and Nestin genes, like it was observed with undifferentiated HMSCs, but now an increase in the GFAP, NF-H, and GAP-43 gene expression is perceived. As indicated in Figure 4, this implies a reduction in Ct values.

### 3.3. Functional Analysis

#### 3.3.1. Nociceptive Function Evaluated by Withdrawal Reflex Latency (WRL)

**Figure 5:**
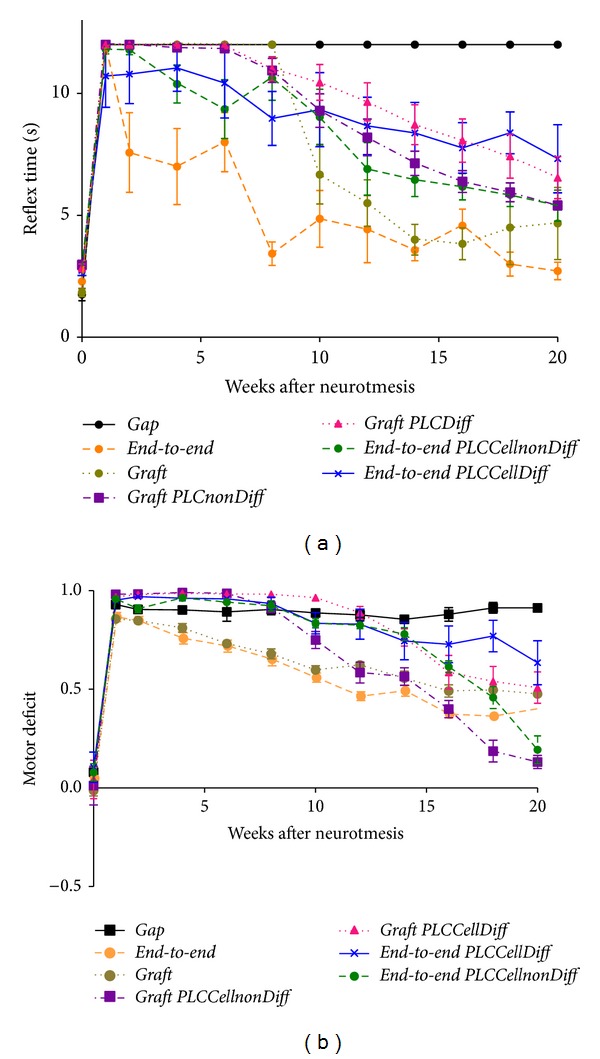
Mean withdrawal reflex latency (WRL) and extensor postural thrust (EPT) results for 20 weeks followup. Values in seconds (s) were obtained performing withdrawal reflex latency (WRL) test to evaluate the nociceptive function (a). Values of motor deficit were obtained performing extensor postural thrust (EPT) test (b). This test has been performed preoperatively (week 0) at week 1, week 2, and every 2 weeks after the surgical procedure until week 20, when the animals were sacrificed for morphological analysis. Data displayed as mean ± SEM.

Figure 5(a) and Table 3 (see Supplementary Material available online at http://dx.doi.org/10.1155/2014/302659) present the data for the WRL during the healing period of 20 weeks. As expected, during the first week (week 1) following sciatic nerve transection and repair, animals were unable to respond to the hot stimulus, indicating complete loss of thermal and nociceptive sensitivity at the sole of the foot. Signs of recovery of foot's withdrawal response began at week 2 following the sciatic nerve injury. Thereafter, the WRL steadily improved during the 20-week recovery time although without recovering its normal value of less than 4 seconds (simple contrasts, 20 weeks versus pre-injury; *P* < 0.05). Anyway, the WRL at week 20 for the treated groups was almost in the normal value range. At week 20, the mean WRL of the* End-to-EndPLCCellnonDif* and* End-to-EndPLCCellDif* group was 5.40 ± 1.56 and 7.32 ± 3.43 seconds, respectively. Concerning the graft groups where the PLC membrane associated to the cellular system the WRL mean values were 5.41 ± 0.46 and 6.54 ± 2.10 seconds, for* GraftPLCCellnonDif* and* GraftPLCCellDif* group, respectively. Differences in WRL recovery were found both as a result of grafting [*F*_(1,31)_ = 7.765; *P* < 0.01] and of HMSCs application [*F*_(2,31)_ = 14.112; *P* < 0.001]. In particular, WRL recovered faster and better in animals treated with direct end-to-end repair (*End-to-End*,* End-to-EndPLCcellnonDif*, and* End-to-EndPLCcellDif* groups) compared to those receiving a graft (*Graft*,* GraftPLCcellnonDif*, and* GraftPLCcellDif* groups). Also, animals treated with differentiated (*P* < 0.001) and undifferentiated (*P* < 0.005) HMSCs showed slight worst WRL response, compared with animals without cellular treatment. Untreated animals (*Gap* group) showed no signs of recovery of the WRL response during the healing period of 20 weeks (Figure 5(a) and Table 3, supplementary data).

#### 3.3.2. Motor Performance by Measuring Extensor Postural Thrust (EPT)

Immediately following sciatic nerve transection, all animals presented a severe motor deficit demonstrated by a virtual complete loss of EPT response in the affected hind limb (Figure 5(b) and Table 4, supplementary data). During the healing period of 20 weeks, the abnormal EPT response improved to some degree in all surgically treated animals although force output remained diminished at the ending of the followup (simple contrasts, 20 weeks versus preinjury; *P* < 0.001). At week 20, the mean EPT of the* End-to-EndPLCCellnonDif* and* End-to-EndPLCCellDif* group was 0.19 ± 0.17 and 0.64 ± 0.27, respectively. Concerning the graft groups where the PLC membrane associated to the cellular system the WRL mean values were 0.13 ± 0.08 and 0.51 ± 0.19, for* GraftPLCCellnonDif* and* GraftPLCCellDif* group, respectively.

The rate of recovery, but not the extent, of the EPT response during recovery was affected by application of HMSCs cells. [*F*_(2,31)_ = 28.778; *P* < 0.001] but not due to grafting [*F*_(1,31)_ = 0.271; *P* > 0.1, non-significant]. Pairwise comparisons showed differences between EPT responses in animals that did not receive the cellular treatment, compared to those treated with undifferentiated (*P* < 0.05) and differentiated HMSCs (*P* < 0.001). Differences in EPT response between animals treated with undifferentiated and differentiated HMSCs were also significant (*P* < 0.001). An interaction effect on EPT responses could be found involving the type of nerve repair (i.e., direct end-to-end repair or grafting) and the application of HMSCs [*F*_(2,31)_ = 4.910; *P* < 0.05]. Pooled EPT data suggests that undifferentiated HMSCs, but not differentiated HMSCs, enhanced EPT recovery in animals that were specifically treated with autologous sciatic nerve graft, thus helping in minimizing the negative consequences of grafting in functional outcome. Also, data suggests that undifferentiated HMSCs, but not differentiated HMSCs, enhanced EPT recovery in animals that were specifically treated with an end-to-end suture (Figure 5(b) and Table 4, supplementary data).

#### 3.3.3. Kinematic Analysis

**Figure 6:**
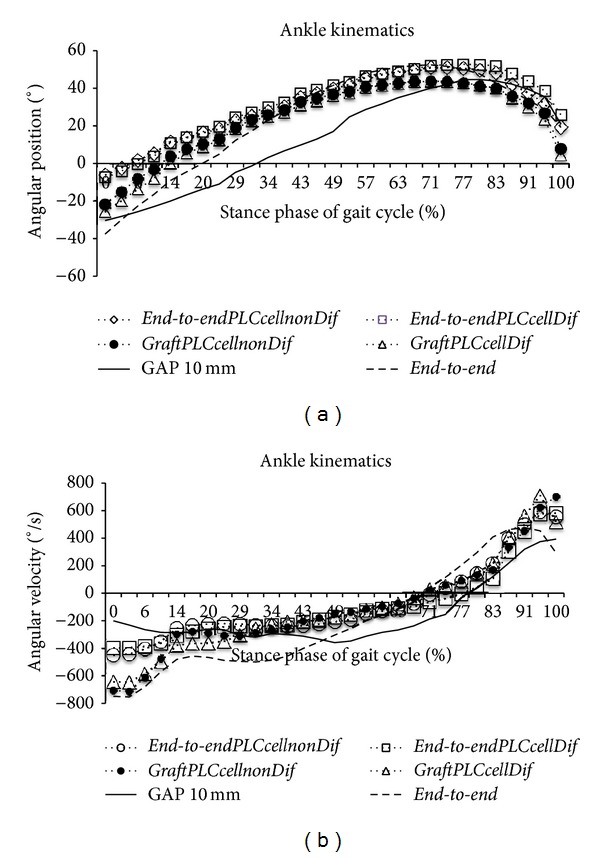
Kinematic plots in the sagittal plane for angular position (°) (a) and for angular velocity (°/s) (b) as it moves through the stance phase, obtained at week 20 after the neurotmesis injury. The mean of each group is plotted (*N* = 6).

The angle and angular velocity of the ankle joint (Figures 6(a) and 6(b) and Table 2) during the stance phase of the step cycle were measured at the end of the study (week 20). These measures were collected also in the uninjured control rats. Regarding sciatic-injured animals, no changes in ankle angle could be seen between the different sciatic nerve-treated experimental groups and between treated groups and the control group, irrespectively of the specific time instant of the stance phase considered (Figure 6(a), Table 2).

Regarding ankle angular velocity, differences between the experimental groups could be found at HR [*F*_(6,33)_ = 10.414; *P* < 0.001] and TO [*F*_(6,33)_ = 2.542; *P* < 0.05] but not at IC [*F*_(6,33)_ = 1.311; *P* > 0.1, non-significant] and OT [*F*_(6,33)_ = 1.776; *P* > 0.1, non-significant]. At HR, ankle velocity was significantly altered in the* Gap* group compared with the* End-to-EndPLCCellDif*,* GraftPLCCellnonDif,* and* GraftPLCCellDif* groups (*P* < 0.05). Also at HR, significant changes in ankle velocity could be seen for every group of sciatic nerve-injured animals compared with intact animals. Similar results were found at TO, where ankle velocity in sciatic nerve-injured animals differed from that of intact animals. However, at TO ankle velocity was similar in every group of sciatic nerve-injured animals (Figure 6(b), Table 2).

### 3.4. Sciatic Nerve Histology

**Figure 7:**
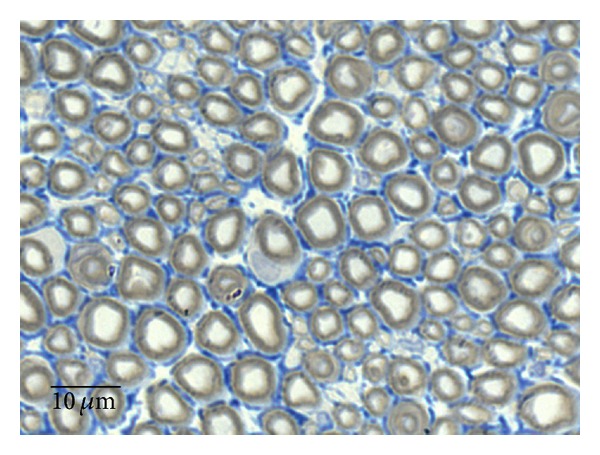
Histological appearance of a normal rat sciatic nerve (magnification: 1000x).

**Figure 8:**
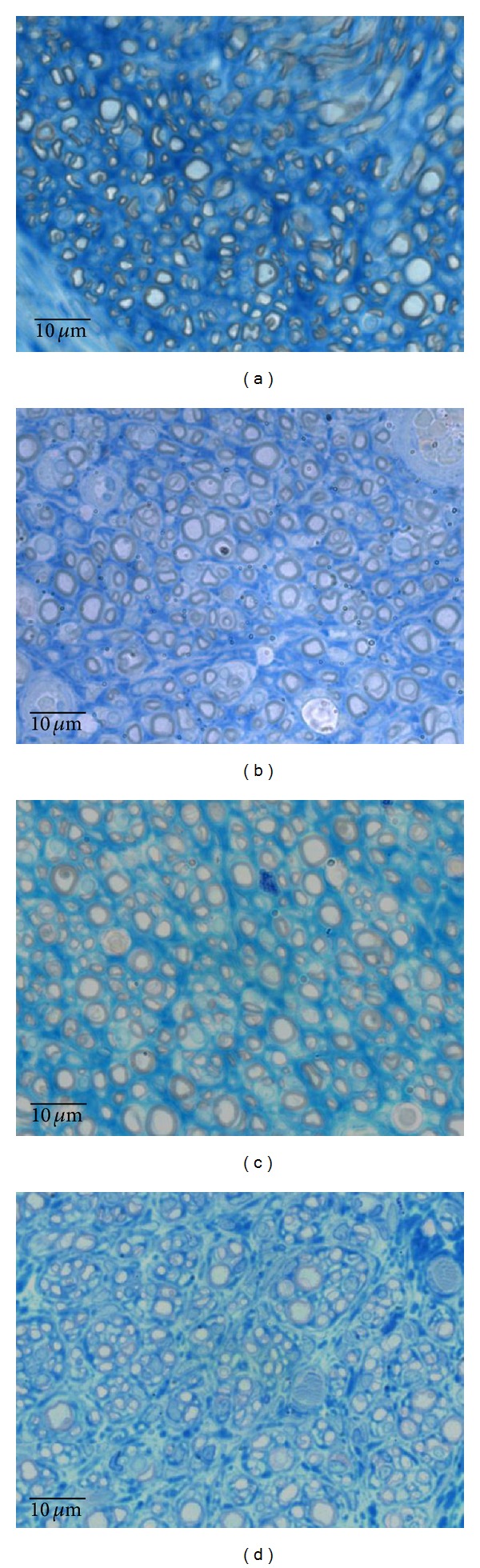
Histological appearance of regenerated nerve fiber treated with undifferentiated and differentiated HMSC:* End-to-endPLCcellnonDif* (a),* End-to-endPLCcellDif* (b),* GraftPLCcellnonDif* (c), and* GraftPLCcellDif* (d) (magnification: 1000x).

Histological analysis on semithin sections showed that nerve fiber regeneration occurred in all repaired nerves. In comparison to controls (Group 1,* Control*) (Figure 7), in all repaired nerves regenerated fibers showed small axons with thin myelin sheaths and microfasciculation (Figures 8(a), 8(b), 8(c), and 8(d)). Microfasciculation was more evident in the graft repaired groups (*GraftPLCCellnonDif* and* GraftPLCCellDif*) (Figures 8(c) and 8(d)) in comparison to* End-to-End* treated groups (*End-to-EndPLCCellnonDif* and* End-to-EndPLCCellDif*) (Figures 8(a) and 8(b)). From a histological point of view, the comparison between treatment with undifferentiated and differentiated HMSC did not show clear differences both after end-to-end (Figures 8(a) and 8(b)) and graft (Figures 8(c) and 8(d)) nerve reconstruction.

## 4. Discussion

Tissue engineering focusing on the* in vitro* fabrication of autologous, living tissues with the potential of regeneration is a promising scientific and clinical field. Peripheral nerve regeneration should include a multidisciplinary team able to develop biomaterials, to develop cell therapies, and to elaborate* in vitro* analysis and preclinical trials concerning animal welfare and the most appropriate animal model before the clinical trials and clinical application approval [46]. Transected peripheral nerves can regenerate spontaneously providing the connection between the proximal and distal severed stumps. In cases where there is no substantial nerve tissue loss, surgical treatment consists in direct end-to-end suturing of the nerve ends [47–51]. However, in spite of the progress achieved with microsurgical nerve repair, the outcome of nerve reconstruction is still far from being optimal^,^ concerning in most of the clinical cases, a poor functional recovery [52]. Since during the regeneration process, axons require neurotrophic support, they could benefit from the presence of a cellular system capable of responding to stimuli of the local environment during axonal regeneration, producing important growth factors and cytokines. In case of loss of substance a nerve autograft procedure, usually using expendable sensory nerves, is required [53–55]. However, the nerve autograft leads to donor site morbidity and secondary sensory deficit and occasionally neuroma and pain. In addition, no donor and recipient nerve diameters match often occurs and the fact of using in most clinical situations, a sensory nerve to reconstruct a motor or a motor and sensory nerve, might be the basis for poor functional recovery [5, 56]. Alternatives to peripheral nerve grafts include cadaver nerve segments allografts, end-to-side neurorrhaphy, or entubulation by means of autologous nonnervous tissues such as vein or muscles [54, 57–61]. Therefore, one of the scientific challenges in the past thirty years has been to find an alternative to the autologous nerve graft [60]. The use of a nerve conduit (i.e., a tubular structure designed to bridge the gap of a sectioned nerve, protect the nerve from the surrounding tissue, and guide the regenerating axons into the distal nerve stump) is the most popular alternative to nerve autografts; yet, conduits can also play an important role as a vehicle for neurotrophic factors and cellular systems [3, 21, 23, 26, 54, 57–59, 62–65].

In a previous study, the therapeutic value of HMSCs on rat sciatic nerve after axonotmesis injury associated to the same PLC membrane was evaluated (Vivosorb). Also,* in vitro* characterization of the cellular system cultured on PLC discs (Vivosorb) was carried out by means of nuclear magnetic resonance (NMR) analysis, immunocytochemistry, and intracellular ionic calcium concentration measurements using the epifluorescence technique. During HMSCs expansion and differentiation into neuroglial-like cells, the analysis of the culture medium, by NMR, was performed in order to evaluate the metabolic profile of these cells. Also, it was necessary to ascertain that PLC membranes were capable of supporting the expansion and differentiation of HMSCs. This was accomplished mainly by assessing [Ca^2+^]_*i*_, using epifluorescence technique and the Fura-2AM probe, of undifferentiated and neuroglial-differentiated HMSCs. The Vivosorb membrane proved to be adequate to be used as scaffold associated with undifferentiated HMSCs or neuroglial-differentiated HMSCs, so that* in vivo* studies could be pursued in models of severe nerve injury, such as in the present study [19]. The same results were obtained in the present experimental work, considering the [Ca^2+^]_*i*_, measurements using epifluorescence technique with Fura-2AM probe.

In this study,* in vitro* results demonstrated successfully the HSMCs differentiation into neuroglia-like cells, as demonstrated by changes in cell morphology and positive staining for the specific neuroglial markers GFAP, GAP-43, and NeuN. Previously, it was demonstrated by NMR, that HMSCs expansion was glycolysis-dependent but that the differentiation of these cells required the switch of the metabolic profile to oxidative metabolism. Simultaneously,* in vivo* studies in a sciatic nerve crush rat model showed improvement in regenerated sciatic nerve's morphology, such as increased myelin sheath thickness, in animals treated with transplanted undifferentiated and differentiated HMSCs, which was accompanied also by enhanced recovery of motor and sensory function [19].

Peripheral nerve crush injuries are appropriate to investigate the cellular and molecular mechanisms of peripheral nerve regeneration and to assess the role of different factors in the regeneration process [66]. Nerve crush injury is also a well-established model in experimental regeneration studies to investigate the impact of various pharmacological treatments [26, 67, 68] and should be used before testing therapeutic approaches in a more serious lesion, like neurotmesis. The present study intended to confirm the ability of PLC membranes together with undifferentiated and* in vitro* differentiated HMSCs to promote nerve regeneration and to improve functional recovery even when nerves are surgically treated by epineural end-to-end suture or autologous grafting. Nerve histology demonstrated successful nerve regeneration of the transected and repaired sciatic nerves although the extent of such regeneration was somewhat limited. Regenerated axons and nerve fibers were small in diameter and their number was clearly diminished in comparison with uninjured nerves. Not surprisingly, such changes in morphology were more severe in sciatic nerves treated with the autologous graft and the cellular system (*GraftPLCCellnonDif* and* GraftPLCCellDif* groups) compared with the direct end-to-end suture and the cellular system (*End-to-EndPLCCellnonDif* and* End-to-EndPLCCellDif*). The use of PLC membranes enwrapping the repaired sciatic nerves could not significantly alter the degree of nerve regeneration. Similarly, coating the sciatic nerves at the injury site with undifferentiated or differentiated HMSCs could not significantly modify the extent of nerve regeneration. These results should be confirmed by histomorphometric analysis, which unfortunately was not possible to be performed in this study. The results confirmed the results obtained previously by Gärtner et al., 2013 [19], where the myelin sheath was thicker in the regenerated nerves of HMSCs-treated animals, suggesting that HMSCs might exert their positive effects on Schwann cells, the key element in Wallerian degeneration and consequent axonal regeneration and explaining the functional recovery improvement, obtained also in the present work. As a matter of fact, regarding functional outcome, the use of undifferentiated HMSCs modestly improved recovery of motor function in the affected hind-limb when in those cases an autologous graft was used to bridge the gap of the transected sciatic nerve. At this point, the mechanisms by which undifferentiated, but not differentiated, HMSCs might enhance functional recovery are not identifiable. For that reason, experiments of RT-PCR were performed with undifferentiated and* in vitro* differentiated HMSCs in this experimental work. According to the results of RT-PCR, undifferentiated HMSCs cells* in vitro* secreted several factors that can aid in nerve regeneration. Consistent with the immunocytochemistry observations in which the antibodies did not recognize GFAP, NF-H, and GAP-43 proteins in the undifferentiated HMSCs in the slides, the molecular analysis showed a small expression of GFAP gene and absence of expression of the NF-H and GAP-43 genes in these same undifferentiated cells. In fact, the small detection of the GFAP gene expression may be due to the high sensitivity of the molecular tests in comparison with immunocytochemistry tests. Moreover, the expression of the remaining genes, NeuN, *β*-actin, GAPDH, and Nestin, was also observed in undifferentiated HMSCs. The expression of the housekeeping genes, *β*-actin and GAPDH, is expected to occur. As per the NeuN and Nestin gene, the observation of its expression in undifferentiated HMSCs is not new; Bertani et al. [69] showed that naïve MSCs express at a constitutive level NeuN gene, which increases when these cells are chemically induced to differentiate to preneuronal cells. Furthermore, Woodbury et al. [70] compared gene expression profiles before and after MSC induction for a number of germ layers and observed that even before neuronal induction, MSC population, and clonal lines expressed a mixture of mesodermal, germinal, endodermal, and ectodermal genes, including several genes whose expression was thought to be restricted to neuronal cells. Molecular analysis on these same genetic markers over the differentiated HMSCs showed an increase in the expression of GFAP, NF-H, and GAP-43 genes. These genes were not expressed, or expressed at very low levels, in the undifferentiated HMSC's transcriptome. Overall, these results support the effective* in vitro* differentiation of HMSCs into neuron-like cells.

HMSCs isolated from Wharton's jelly of the umbilical cord and delivered through PLC membranes might improve clinical outcome especially after trauma to sensory nerves, particularly in the cases of nerve injuries with significant loss of nervous tissue, requiring entubulation or grafting. When the neurotmesis injury can be surgically reconstructed with an epineural end-to-end suture without tension or by grafting, the addition of a PLC membrane associated with undifferentiated HMSCs seems to bring significant advantage, especially concerning the motor function recovery, basically by the secretion of local growth factors and cytokines secretion.

## Supplementary Material

Table 3 represents values in seconds (s) that were obtained performing Withdrawal Reflex Latency (WRL) test to evaluate the nociceptive function. This test has been performed pre-operatively (week-0), at week 1 and 2 and after every two weeks until the end of the 20-week follow-up time. Results are presented as mean and standard deviation (SD). N corresponds to the number of rats within the experimental group.
Table 4 represents values of Motor Deficit that were obtained performing Extensor Postural Thrust (EPT) test. This test has been performed pre-operatively (week-0), at week 1 and 2 and after every two weeks until the end of the 20-week follow-up time. Results are presented as mean and standard deviation (SD). N corresponds to the number of rats within the experimental group.

## Figures and Tables

**Table 1:** List of primers used, target gene, and size of the PCR product.

Primer	GenBank target gene	PCR product
GFAP hum Fwd: 5′-CCAGCTGCGGGCCAAGGA-3′	NG_008401.1	56 bp
GFAP hum Rev: 5′-GCAGCTCAGCCTGGTAGACG-3′		

NeuN hum Fwd: 5′-AGTAGCTGGGAATTATGGA-3′	NM_005598.3	157 bp
NeuN hum Rev: 5′-ATTGGGACAGTAGGAGTCAGA-3′		

*β*-actin hum Fwd: 5′-GGCACCCAGCACAATGAAGA-3′	NM_001101.3	100 bp
*β*-actin hum Rev: 5′-CTGGAAGGTGGACAGCGAGGC-3′		

GAPDH hum Fwd: 5′-CCCTGCCTCTACTGGCGC-3′	XM_005253678.1	60 bp
GAPDH hum Rev: 5′-TTCCCGTTCAGCTCAGGG-3′		

Nestin hum Fwd: 5′-GGCAGCGTTGGAACAGAGGTTGGA-3′	NM_006617.1	164 bp
Nestin hum Rev: 5′-ACATCTTGAGGTGCGCCAGCT-3′		

NF-H hum Fwd: 5′-GTGGTGGAGAAGTCTGAGAA-3′	NM_021076.3	176 bp
NF-H hum Rev: 5′-GGAGACTTTGTTTCTTCTTC-3′		

GAP-43 hum Fwd: 5′-TGCTGTGCTGTATGAGAAGAACC-3′	NM_001130064.1	160 bp
GAP-43 hum Rev: 5′-GCAAGGGCTGAGGTGTTATGA-3′		

**Table 2:** Kinematic data for angular position (°) and for angular velocity (°/s) as it moves through the stance phase, obtained at week 20 after the neurotmesis injury.

	Initial contact		Opposite toe off		Heel rise		Toe off	
	Position (°)	Velocity (°/s)	Position (°)	Velocity (°/s)	Position (°)	Velocity (°/s)	Position (°)	Velocity (°/s)
*Control *	−11.7 (7.3)	−405.9 (303.2)	27.0 (9.0)	−353.8 (234.9)	38.5 (10.0)	−48.3 (103.5)	9.5 (19.6)	51.1 (413.1)
*End-to-end *	−39.3 (12.1)	−835.7 (249.2)	20.9 (8.9)	−376.9 (109.4)	38.8 (7.2)	−267.4 (56.0)∗	13.2 (10.7)	437.2 (214.8)∗
*Gap *	−31.8 (5.4)	−447.6 (464.6)	14.1 (2.7)	−494.7 (204.2)	31.4 (5.8)	−374.3 (30.6)∗	17.1 (14.5)	392.7 (328.8)∗
*End-to-end PLCCellnonDiff *	−6.0 (13.0)	−450.0 (191.9)	16.2 (14.7)	−223.8 (94.0)	32.6 (13.3)	−239.5 (41.6)∗	19.3 (8.8)	556.0 (195.2)∗
*End-to-end PLCCellDiff *	−7.2 (11.5)	−396.6 (354.2)	16.9 (11.3)	−257.6 (88.5)	34.8 (9.9)	−223.7 (50.8)^∗†^	25.9 (17.8)	589.2 (316.2)∗
*GraftPLCcellnonDif *	0.5 (51.4)	−630.0 (249.9)	16.8 (13.0)	−269.3 (45.5)	35.5 (12.4)	−198.0 (69.7)^∗†^	−14.0 (48.2)	508.3 (175.0)∗
*GraftPLCCellDiff *	−25.6 (20.7)	−643.5 (281.2)	10.8 (14.5)	−356.5 (314.0)	30.1 (15.7)	−200.9 (85.0)^∗†^	1.3 (22.1)	343.4 (248.4)∗

The mean (and SD) values of each group are described in the table (*N* = 6). ∗Significantly different from control (*P* < 0.05). ^†^Significantly different from neurogap (*P* < 0.05).
