# Supplementary material for: Promoting Nerve Regeneration in a Neurotmesis Rat Model Using Poly(DL-lactide-ε-caprolactone) Membranes and Mesenchymal Stem Cells from the Wharton's Jelly: In Vitro and In Vivo Analysis
Source: Biomed Res Int. 2014 Jul 10;2014:302659. doi: 10.1155/2014/302659 (PMC4119891; doi:10.1155/2014/302659)
Supplement: Supplementary file 1 — Table 3 represents values in seconds (s) that were obtained performing Withdrawal Reflex Latency (WRL) test to evaluate the nociceptive function. This test has been performed pre-operatively (week-0), at week 1 and 2 and after every two weeks until the end of the 20-week follow-up time. Results are presented as mean and standard deviation (SD). N corresponds to the number of rats within the experimental group. Table 4 represents values of Motor Deficit that were obtained performing Extensor Postural Thrust (EPT) test. This test has been performed pre-operatively (week-0), at week 1 and 2 and after every two weeks until the end of the 20-week follow-up time. Results are presented as mean and standard deviation (SD). N corresponds to the number of rats within the experimental group. [file 302659.f1.docx]

**Supplementary data**

**Table 3 -** Values in seconds (s) were obtained performing Withdrawal Reflex Latency (WRL) test to evaluate the nociceptive function. This test has been performed pre-operatively (week-0), at week 1 and 2 and after every two weeks until the end of the 20-week follow-up time. Results are presented as mean and standard deviation (SD). N corresponds to the number of rats within the experimental group.

|  | **Week 0** | **Week 1** | **Week 2** | **Week 4** | **Week 6** | **Week 8** | **Week 10** | **Week 12** | **Week 14** | **Week 16** | **Week 18** | **Week 20** |
| --- | --- | --- | --- | --- | --- | --- | --- | --- | --- | --- | --- | --- |
| Gap |  |  |  |  |  |  |  |  |  |  |  |  |
| **1A** | 2,00 | 12,00 | 12,00 | 12,00 | 12,00 | 12,00 | 12,00 | 12,00 | 12,00 | 12,00 | 12,00 | 12,00 |
| **1B** | 1,00 | 12,00 | 12,00 | 12,00 | 12,00 | 12,00 | 12,00 | 12,00 | 12,00 | 12,00 | 12,00 | 12,00 |
| **1C** | 2,00 | 12,00 | 12,00 | 12,00 | 12,00 | 12,00 | 12,00 | 12,00 | 12,00 | 12,00 | 12,00 | 12,00 |
| **1D** | 2,00 | 12,00 | 12,00 | 12,00 | 12,00 | 12,00 | 12,00 | 12,00 | 12,00 | 12,00 | 12,00 | 12,00 |
| Mean ± SD | 1,75±0,50 | 12,00±0,00 | 12,00±0,00 | 12,00±0,00 | 12,00±0,00 | 48,00±0,00 | 12,00±0,00 | 12,00±0,00 | 12,00±0,00 | 12,00±0,00 | 12,00±0,00 | 12,00±0,00 |
| End-to-end |  |  |  |  |  |  |  |  |  |  |  |  |
| **2A** | 3,00 | 12,00 | 4,00 | 4,00 | 10,00 | 5,00 | 8,00 | 12,00 | 5,00 | 7,00 | 5,00 | 4,00 |
| **2B** | 2,00 | 12,00 | 5,00 | 12,00 | 5,00 | 2,00 | 10,00 | 2,00 | 4,00 | 2,00 | 2,00 | 3,00 |
| **2C** | 2,00 | 12,00 | 12,00 | 8,00 | 5,00 | 3,00 | 4,00 | 4,00 | 4,00 | 6,00 | 4,00 | 4,00 |
| **2D** | 4,00 | 12,00 | 2,00 | 3,00 | 4,00 | 5,00 | 2,00 | 6,00 | 4,00 | 4,00 | 4,00 | 2,00 |
| **2E** | 2,00 | 12,00 | 6,00 | 2,00 | 10,00 | 2,00 | 5,00 | 2,00 | 2,00 | 6,00 | 2,00 | 2,00 |
| **2F** | 2,00 | 12,00 | 12,00 | 12,00 | 12,00 | 3,00 | 3,00 | 3,00 | 4,00 | 4,00 | 2,00 | 2,00 |
| **2G** | 1,00 | 12,00 | 12,00 | 8,00 | 10,00 | 4,00 | 2,00 | 2,00 | 2,00 | 3,00 | 2,00 | 2,00 |
| Mean ± SD | 2.29±0.95 | 12.0±0.00 | 7.57±4.31 | 7.00±4.12 | 8.00±3.21 | 3.43±1.27 | 4.86±3.08 | 4.43±3.64 | 3.57±1.13 | 4.57±1.81 | 3.00±1.29 | 2.71±0.95 |
| Graft | | | | | | | | | |  |  |  |
| **3A** | 2,00 | 12,00 | 12,00 | 12,00 | 12,00 | 12,00 | 5,00 | 4,00 | 3,00 | 4,00 | 4,00 | 4,00 |
| **3B** | 2,00 | 12,00 | 12,00 | 12,00 | 12,00 | 12,00 | 8,00 | 5,00 | 3,00 | 3,00 | 3,00 | 3,00 |
| **3C** | 2,00 | 12,00 | 12,00 | 12,00 | 12,00 | 12,00 | 4,00 | 4,00 | 3,00 | 3,00 | 3,00 | 3,00 |
| **3D** | 2,00 | 12,00 | 12,00 | 12,00 | 12,00 | 12,00 | 12,00 | 10,00 | 7,00 | 7,00 | 12,00 | 12,00 |
| **3E**  **3F** | 1,00 | 12,00 | 12,00 | 12,00 | 12,00 | 12,00 | 6,00 | 6,00 | 4,00 | 3,00 | 3,00 | 3,00 |
|  | 2,00 | 12,00 | 12,00 | 12,00 | 12,00 | 12,00 | 5,00 | 4,00 | 4,00 | 3,00 | 2,00 | 3,00 |
| Mean ± SD | 1,83±0,41 | 12,00±0,00 | 12,00±0,00 | 12,00±0,00 | 12,00±0,00 | 12,00±0,00 | 6,67±2,94 | 5,50±2,35 | 4,00±1,55 | 3,83±1,60 | 4,50±3,73 | 4,67±3,61 |
| End-to-end PLCCellnonDif | | | |  |  |  |  |  |  |  |  |  |
| **4A** | 2,89 | 12,00 | 12,00 | 12,00 | 11,05 | 12,00 | 12,00 | 9,34 | 7,29 | 7,58 | 5,64 | 3,92 |
| **4B** | 3,08 | 10,86 | 10,77 | 8,44 | 7,35 | 7,10 | 5,64 | 5,44 | 5,03 | 4,24 | 4,10 | 3,84 |
| **4C** | 3,25 | 12,00 | 12,00 | 7,88 | 9,12 | 8,59 | 8,02 | 7,29 | 7,40 | 6,55 | 6,49 | 5,92 |
| **4D** | 3,00 | 12,00 | 12,00 | 12,00 | 12,00 | 12,00 | 12,00 | 5,26 | 8,03 | 7,61 | 7,65 | 7,80 |
| **4E** | 2,74 | 12,00 | 12,00 | 10,00 | 4,60 | 12,00 | 6,40 | 3,58 | 3,71 | 5,34 | 5,88 | 6,31 |
| **4F** | 2,40 | 12,00 | 12,00 | 12,00 | 12,00 | 12,00 | 10,15 | 10,49 | 7,26 | 5,73 | 5,30 | 4,60 |
| Mean ± SD | 2,89±0,30 | 11,81±0,47 | 11,80±0,50 | 10,39±1,90 | 9,35±2,95 | 10,62±2,20 | 9,04±2,77 | 6,90±2,64 | 6,45±1,69 | 6,18±1,33 | 5,84±1,19 | 5,40±1,56 |
| End-to-end PLCCellDif | | | | | | |  |  |  |  |  |  |
| **5A** | 3,03 | 12,00 | 12,00 | 12,00 | 12,00 | 9,75 | 12,00 | 9,49 | 9,81 | 7,99 | 9,35 | 3,87 |
| **5B** | 2,21 | 4,29 | 4,75 | 6,26 | 3,29 | 4,83 | 3,94 | 3,62 | 4,72 | 6,28 | 7,75 | 4,34 |
| **5C** | 2,19 | 12,00 | 12,00 | 12,00 | 12,00 | 6,68 | 12,00 | 9,33 | 9,72 | 5,79 | 8,31 | 11,54 |
| **5D** | 3,06 | 12,00 | 12,00 | 12,00 | 12,00 | 11,06 | 10,84 | 10,11 | 9,60 | 9,20 | 8,19 | 6,75 |
| **5E** | 2,54 | 12,00 | 12,00 | 12,00 | 11,27 | 9,51 | 5,24 | 7,45 | 4,43 | 5,32 | 5,13 | 5,90 |
| **5F** | 3,42 | 12,00 | 12,00 | 12,00 | 12,00 | 12,00 | 12,00 | 12,00 | 12,00 | 12,00 | 11,56 | 11,54 |
| Mean ± SD | 2,74±0,50 | 10,72±3,15 | 10,79±2,96 | 11,04±2,34 | 10,43±3,51 | 8,97±2,71 | 9,34±3,73 | 8,67±2,87 | 8,38±3,08 | 7,76±2,53 | 8,38±2,10 | 7,32±3,43 |
| Graft PLCCellnonDif | | | | | | |  |  |  |  |  |  |
| **6A** | 2,88 | 12,00 | 12,00 | 11,27 | 11,06 | 11,04 | 10,39 | 9,02 | 8,77 | 8,19 | 7,64 | 6,20 |
| **6B** | 3,03 | 12,00 | 12,00 | 12,00 | 12,00 | 12,00 | 11,74 | 11,14 | 6,44 | 5,60 | 5,88 | 5,29 |
| **6C** | 3,36 | 12,00 | 12,00 | 12,00 | 12,00 | 9,54 | 8,24 | 6,67 | 6,34 | 6,13 | 5,73 | 5,53 |
| **6D** | 2,87 | 12,00 | 12,00 | 12,00 | 12,00 | 11,96 | 8,81 | 7,37 | 7,47 | 6,56 | 5,77 | 5,38 |
| **6E** | 2,18 | 12,00 | 12,00 | 12,00 | 12,00 | 11,75 | 9,66 | 8,82 | 8,20 | 6,82 | 5,93 | 5,24 |
| **6F** | 3,48 | 12,00 | 12,00 | 12,00 | 12,00 | 9,35 | 6,92 | 6,16 | 5,58 | 5,02 | 4,72 | 4,80 |
| Mean ± SD | 2,97±0,46 | 12,00±0,00 | 12,00±0,00 | 11,88±0,30 | 11,84±0,38 | 10,94±1,21 | 9,29±1,69 | 8,20±1,84 | 7,13±1,22 | 6,39±1,10 | 5,59±0,94 | 5,41±0,46 |
| Graft PLCCellDif | | | | | | |  |  |  |  |  |  |
| **7A** | 3,12 | 12,00 | 12,00 | 12,00 | 12,00 | 12,00 | 11,74 | 10,17 | 9,05 | 8,22 | 7,15 | 5,21 |
| **7B** | 3,50 | 12,00 | 12,00 | 12,00 | 11,86 | 9,64 | 9,45 | 8,72 | 7,85 | 7,31 | 5,98 | 5,58 |
| **7C** | 2,67 | 12,00 | 12,00 | 12,00 | 12,00 | 12,00 | 12,00 | 12,00 | 12,00 | 12,00 | 11,56 | 10,35 |
| **7D** | 2,64 | 12,00 | 12,00 | 12,00 | 12,00 | 10,86 | 10,42 | 9,74 | 8,33 | 6,33 | 6,02 | 5,63 |
| **7E** | 2,52 | 12,00 | 12,00 | 12,00 | 12,00 | 12,00 | 11,73 | 10,81 | 9,18 | 8,51 | 8,04 | 7,61 |
| **7F** | 2,62 | 12,00 | 12,00 | 12,00 | 12,00 | 9,57 | 7,37 | 6,47 | 5,89 | 6,03 | 5,80 | 4,87 |
| Mean ± SD | 2,84±0,38 | 12,00±0,00 | 12,00±0,00 | 12,00±0,00 | 11,98±0,06 | 11,01±1,18 | 10,45±1,80 | 9,65±1,90 | 8,72±2,00 | 8,07±2,16 | 7,43±2,20 | 6,54±2,10 |

**Table 4** - Values of Motor Deficit were obtained performing Extensor Postural Thrust (EPT) test. This test has been performed pre-operatively (week-0), at week 1 and 2 and after every two weeks until the end of the 20-week follow-up time. Results are presented as mean and standard deviation (SD). N corresponds to the number of rats within the experimental group.

|  | **Week 0** | **Week 1** | **Week 2** | **Week 4** | **Week 6** | **Week 8** | **Week 10** | **Week 12** | **Week 14** | **Week 16** | **Week 18** | **Week 20** |
| --- | --- | --- | --- | --- | --- | --- | --- | --- | --- | --- | --- | --- |
| Gap |  |  |  |  |  |  |  |  |  |  |  |  |
| **1A** | 0,08 | 0,90 | 0,92 | 0,92 | 0,92 | 0,98 | 0,87 | 0,84 | 0,84 | 0,91 | 0,96 | 0,94 |
| **1B** | 0,09 | 0,89 | 0,90 | 0,90 | 0,95 | 0,89 | 0,87 | 0,90 | 0,89 | 0,95 | 0,90 | 0,95 |
| **1C** | 0,07 | 0,95 | 0,90 | 0,87 | 0,75 | 0,85 | 0,90 | 0,82 | 0,80 | 0,79 | 0,84 | 0,86 |
| **1D** | 0,08 | 0,98 | 0,90 | 0,92 | 0,95 | 0,90 | 0,91 | 0,95 | 0,89 | 0,87 | 0,95 | 0,90 |
| Mean ± SD | 0,08±0,01 | 0,93±0,04 | 0,91±0,01 | 0,90±0,02 | 0,89±0,10 | 0,91±0,05 | 0,89±0,02 | 0,88±0,06 | 0,86±0,04 | 0,88±0,07 | 0,91±0,06 | 0,91±0,04 |
| End-to-end |  |  |  |  |  |  |  |  |  |  |  |  |
| **2A** | 0,07 | 0,90 | 0,80 | 0,73 | 0,68 | 0,56 | 0,54 | 0,54 | 0,64 | 0,48 | 0,42 | 0,50 |
| **2B** | 0,07 | 0,93 | 0,83 | 0,70 | 0,73 | 0,66 | 0,57 | 0,50 | 0,45 | 0,40 | 0,40 | 0,40 |
| **2C** | 0,08 | 0,90 | 0,85 | 0,88 | 0,82 | 0,76 | 0,57 | 0,50 | 0,53 | 0,44 | 0,38 | 0,40 |
| **2D** | 0,07 | 0,90 | 0,91 | 0,84 | 0,82 | 0,72 | 0,57 | 0,39 | 0,55 | 0,38 | 0,34 | 0,40 |
| **2E** | 0,09 | 0,97 | 0,97 | 0,69 | 0,60 | 0,56 | 0,51 | 0,46 | 0,45 | 0,38 | 0,38 | 0,39 |
| **2F** | 0,06 | 0,77 | 0,86 | 0,83 | 0,78 | 0,72 | 0,63 | 0,57 | 0,55 | 0,38 | 0,38 | 0,40 |
| **2G** | 0,07 | 0,87 | 0,89 | 0,81 | 0,78 | 0,76 | 0,70 | 0,47 | 0,45 | 0,33 | 0,42 | 0,50 |
| Mean ± SD | 0,07±0,01 | 0,89±0,06 | 0,87±0,06 | 0,78±0,08 | 0,74±0,08 | 0,68±0,09 | 0,58±0,06 | 0,49±0,06 | 0,52±0,07 | 0,40±0,05 | 0,39±0,03 | 0,43±0,05 |
| Graft | | | | | | | | | |  |  |  |
| **3A** | 0,07 | 0,83 | 0,87 | 0,78 | 0,78 | 0,81 | 0,73 | 0,72 | 0,69 | 0,60 | 0,50 | 0,50 |
| **3B** | 0,08 | 0,89 | 0,91 | 0,92 | 0,75 | 0,75 | 0,60 | 0,64 | 0,53 | 0,46 | 0,54 | 0,52 |
| **3C** | -0,08 | 0,93 | 0,94 | 0,88 | 0,78 | 0,65 | 0,63 | 0,63 | 0,50 | 0,45 | 0,57 | 0,54 |
| **3D** | 0,00 | 0,91 | 0,85 | 0,82 | 0,77 | 0,72 | 0,58 | 0,68 | 0,64 | 0,54 | 0,57 | 0,54 |
| **3E**  **3F** | 0,09 | 0,93 | 0,89 | 0,82 | 0,77 | 0,74 | 0,63 | 0,64 | 0,69 | 0,64 | 0,50 | 0,54 |
|  | 0,00 | 0,89 | 0,87 | 0,89 | 0,78 | 0,65 | 0,66 | 0,67 | 0,53 | 0,50 | 0,54 | 0,46 |
| Mean ± SD | 0,03±0,07 | 0,90±0,04 | 0,89±0,03 | 0,85±0,05 | 0,77±0,01 | 0,72±0,06 | 0,64±0,05 | 0,66±0,03 | 0,60±0,09 | 0,53±0,08 | 0,54±0,03 | 0,52±0,03 |
| End-to-end PLCCellnonDif | | | |  |  |  |  |  |  |  |  |  |
| **4A** | 0,03 | 0,97 | 0,94 | 0,98 | 0,97 | 0,88 | 0,80 | 0,84 | 0,79 | 0,72 | 0,50 | 0,12 |
| **4B** | 0,06 | 0,97 | 0,98 | 0,98 | 0,99 | 0,99 | 0,85 | 0,85 | 0,80 | 0,61 | 0,45 | 0,50 |
| **4C** | 0,26 | 0,99 | 0,92 | 0,98 | 0,91 | 0,91 | 0,90 | 0,82 | 0,87 | 0,64 | 0,60 | 0,28 |
| **4D** | 0,14 | 0,97 | 0,86 | 0,98 | 0,93 | 0,94 | 0,90 | 0,88 | 0,83 | 0,64 | 0,60 | 0,08 |
| **4E** | 0,03 | 0,95 | 0,88 | 0,88 | 0,90 | 0,83 | 0,77 | 0,76 | 0,75 | 0,57 | 0,29 | 0,15 |
| **4F** | -0,05 | 0,90 | 0,87 | 0,98 | 0,95 | 0,99 | 0,79 | 0,80 | 0,64 | 0,51 | 0,31 | 0,03 |
| Mean ± SD | 0,08±0,11 | 0,96±0,03 | 0,91±0,05 | 0,96±0,04 | 0,94±0,03 | 0,92±0,06 | 0,84±0,06 | 0,83±0,04 | 0,78±0,08 | 0,62±0,07 | 0,46±0,14 | 0,19±0,17 |
| End-to-end PLCCellDif | | | | | | |  |  |  |  |  |  |
| **5A** | 0,13 | 0,95 | 0,97 | 0,97 | 0,97 | 1,00 | 0,98 | 0,99 | 0,98 | 0,99 | 0,99 | 0,87 |
| **5B** | 0,02 | 0,92 | 0,98 | 0,94 | 0,93 | 0,98 | 0,75 | 0,89 | 0,55 | 0,59 | 0,75 | 0,22 |
| **5C** | -0,08 | 0,96 | 0,97 | 0,96 | 0,94 | 0,98 | 0,99 | 0,89 | 0,76 | 0,73 | 0,61 | 0,66 |
| **5D** | 0,10 | 0,98 | 0,98 | 0,99 | 0,98 | 0,83 | 0,86 | 0,70 | 0,80 | 0,68 | 0,50 | 0,48 |
| **5E** | 0,00 | 0,92 | 0,94 | 0,92 | 0,95 | 0,84 | 0,77 | 0,52 | 0,40 | 0,40 | 0,79 | 0,60 |
| **5F** | 0,46 | 0,98 | 0,98 | 0,99 | 0,98 | 0,98 | 0,66 | 0,99 | 0,98 | 0,98 | 0,98 | 0,98 |
| Mean ± SD | 0,11±0,19 | 0,11±0,19 | 0,97±0,02 | 0,96±0,03 | 0,96±0,02 | 0,94±0,08 | 0,84±0,13 | 0,83±0,19 | 0,75±0,23 | 0,75±0,23 | 0,77±0,23 | 0,64±0,27 |
| Graft PLCCellnonDif | | | | | | |  |  |  |  |  |  |
| **6A** | 0,03 | 0,97 | 0,98 | 0,99 | 0,99 | 0,89 | 0,76 | 0,56 | 0,59 | 0,53 | 0,16 | 0,21 |
| **6B** | 0,24 | 0,99 | 0,99 | 0,99 | 0,98 | 0,85 | 0,70 | 0,70 | 0,67 | 0,48 | 0,05 | 0,13 |
| **6C** | -0,02 | 0,99 | 0,98 | 0,99 | 0,99 | 0,94 | 0,72 | 0,67 | 0,48 | 0,40 | 0,32 | 0,23 |
| **6D** | 0,23 | 0,99 | 0,98 | 0,99 | 0,99 | 1,00 | 0,88 | 0,50 | 0,60 | 0,28 | 0,35 | 0,13 |
| **6E** | 0,01 | 0,97 | 0,98 | 0,99 | 0,99 | 0,84 | 0,59 | 0,38 | 0,39 | 0,27 | 0,22 | 0,08 |
| **6F** | -0,42 | 0,98 | 0,99 | 0,99 | 0,98 | 0,98 | 0,85 | 0,70 | 0,66 | 0,44 | 0,02 | 0,01 |
| Mean ± SD | 0,01±0,24 | 0,98±0,01 | 0,98±0,01 | 0,99±0,00 | 0,99±0,01 | 0,92±0,07 | 0,75±0,11 | 0,59±0,13 | 0,57±0,11 | 0,40±0,11 | 0,19±0,14 | 0,13±0,08 |
| Graft PLCCellDif | | | | | | |  |  |  |  |  |  |
| **7A** | -0,15 | 0,90 | 0,98 | 0,99 | 0,98 | 0,98 | 0,94 | 0,84 | 0,73 | 0,64 | 0,60 | 0,53 |
| **7B** | 0,18 | 0,98 | 0,98 | 0,99 | 0,99 | 0,98 | 0,98 | 0,93 | 0,77 | 0,49 | 0,38 | 0,35 |
| **7C** | 0,15 | 0,98 | 0,97 | 0,98 | 0,98 | 0,98 | 0,98 | 0,94 | 0,92 | 0,87 | 0,76 | 0,77 |
| **7D** | -0,06 | 0,99 | 0,98 | 0,99 | 0,98 | 0,98 | 0,99 | 0,98 | 0,85 | 0,69 | 0,63 | 0,66 |
| **7E** | 0,39 | 0,98 | 0,98 | 0,98 | 0,98 | 0,98 | 0,98 | 0,88 | 0,63 | 0,58 | 0,61 | 0,50 |
| **7F** | -0,25 | 0,98 | 0,99 | 0,99 | 0,99 | 0,99 | 0,92 | 0,76 | 0,68 | 0,25 | 0,26 | 0,24 |
| Mean ± SD | 0,24±0,04 | 0,97±0,03 | 0,98±0,01 | 0,99±0,01 | 0,98±0,01 | 0,98±0,00 | 0,97±0,03 | 0,89±0,08 | 0,76±0,11 | 0,59±0,21 | 0,54±0,18 | 0,51±0,19 |
